# Feasibility of a *Lactobacillus casei* Drink in the Intensive Care Unit for Prevention of Antibiotic Associated Diarrhea and *Clostridium difficile*

**DOI:** 10.3390/nu10050539

**Published:** 2018-04-26

**Authors:** Cathy Alberda, Sam Marcushamer, Tayne Hewer, Nicole Journault, Demetrios Kutsogiannis

**Affiliations:** 1 Alberta Health Services, 670 CSC Royal Alexandra Hospital, Edmonton, AB T5H 3V9, Canada; nicole.journault@ahs.ca; 2 Department of Critical Care Medicine, Faculty of Medicine and Dentistry, Edmonton, AB T5H 3V9, Canada; smarcush@ualberta.ca (S.M.); djk3@ualberta.ca (D.K.); 3 Department of Medicine, Faculty of Medicine and Dentistry, University of Alberta, Edmonton, AB T5H 3V9, Canada; tayne.hewer@ahs.ca

**Keywords:** ICU, diarrhea, *Clostridium difficile*, *Lactobacillus casei* drink

## Abstract

**Background:** Over 70% of patients are prescribed antibiotics during their intensive care (ICU) admission. The gut microbiome is dramatically altered early in an ICU stay, increasing the risk for antibiotic associated diarrhea (AAD) and *Clostridium difficile* infections (CDI). Evidence suggests that some probiotics are effective in the primary prevention of AAD and CDI. **Aim:** To demonstrate safety and feasibility of a probiotic drink in ICU patients. **Methods:** ICU patients initiated on antibiotics were recruited, and matched with contemporary controls. Study patients received two bottles daily of a drink containing 10 billion *Lactobacillus casei* which was bolused via feeding tube. Tolerance to probiotics and enteral nutrition, development of adverse events, and incidence of AAD was recorded. CDI rates were followed for 30 days post antibiotic treatment. **Results:** Thirty-two patients participated in the trial. There were no serious adverse events in the probiotic group, compared to three in the control group. AAD was documented in 12.5% of the probiotic group and 31.3% in the control group. One patient in the probiotic group developed CDI compared to three in the control group. **Discussion:** A probiotic containing drink can safely be delivered via feeding tube and should be considered as a preventative measure for AAD and CDI in ICU.

## 1. Background

Antibiotics are amongst the most prescribed medications worldwide, and are particularly common place in the Intensive Care Unit (ICU). The US Centers for Disease Control indicates that 55% of all hospitalized patients receive at least one antibiotic during their hospital stay. In ICU, this number increases to greater than 70%, often with multiple antibiotics [1]. Antibiotic treatment can disturb the colonization resistance of gastrointestinal flora resulting in antibiotic-associated diarrhea (AAD) caused by pathogenic bacterial overgrowth [2]. Of the patients who develop AAD, approximately 1/3 will develop *Clostridium difficile* infection (CDI) [2]. *Clostridium difficile* toxin is the pathogen most often associated with adverse events in the ICU. In Canada, hospital acquired CDI increases healthcare costs four-fold [3]. In addition, one of every ten patients who acquires CDI in Canadian hospitals will die [4]. McDonald et al. has demonstrated that the microbiome of patients admitted to the ICU differs dramatically from that of healthy patients [5]. Treatments used in the ICU, including antibiotics, low blood flow to the gut, together with lack of enteral nutrition can reduce the population of known healthy bacteria. The change from a normal healthy gut microbiota consisting largely of Firmicutes and Bacteroidetes bacteria changes within days to a gut microbiota predominated by Proteobacteria, which includes many pathogens [5]. Interventions that target restoration of bacterial balance could reduce the risk of infection by dangerous pathogens, including CDI.

Certain populations are at higher risk for the development of AAD. The prevalence of diarrhea in ICU has been reported to be as high as 68% [6]. Anyone who has taken antibiotics within the past month is at risk with a documented prevalence of 5–39% in adults and 15–20% in the elderly [7].

In addition, certain antibiotics have been identified as “high risk” for development of AAD and CDI [8]. These antibiotics include the Cephalosporins, Penicillins and Fluoroquinolones, all of which are commonplace in the ICU. The severity of CDI is variable with abdominal pain and/or distention in its mildest form to intractable diarrhea, toxic megacolon, and death in its most severe forms. The financial burden of CDI is significant.

Probiotics are microorganisms that are believed to counteract disturbances in intestinal flora, thus reducing its risk of colonization by pathogenic bacteria [9,10]. With increasing ICU antibacterial resistance rates and fewer new antibiotics emerging, attention has been shifted to non-antibiotic approaches for the prevention and treatment of nosocomial infections [11]. CDI incidence and severity in high income countries has increased dramatically over the past decade [12]. Probiotic benefits to ICU patients may include the prevention of AAD and CDI. Current probiotic research is complicated by the heterogeneity of probiotic strains, doses and treatment durations. However, a 2013 systematic review of this literature, which included 31 randomized trials with a total of 4492 participants, offers moderate quality evidence suggesting that prophylactic probiotics are both safe and effective for preventing *Clostridium difficile* associated diarrhea [13]. These results were confirmed by a 2017 systematic review [14]. A conflicting study found no benefit to probiotics in prevention of AAD and *C. difficile*, however, the probiotic in that study was provided up to seven days after antibiotics were initiated [15]. Studies that demonstrate the most promise with prevention of AAD and probiotics have used *Lactobacillus casei* and *Lactobacillus acidophilus* provided within 48 h of the antibiotic initiation [16]. Researchers in Quebec have demonstrated a decline in CDI rates from 18.0 cases per 10,000 patient-days to 2.3 cases per 10,000 patient-days when a mixture of Lactobacilli was implemented along with standard protective measures [17].

The efficacy of a probiotic containing food on AAD and CDI rates is less well documented in the literature. Hickson showed a 65% lower hospital acquired CDI rate in hospitalized patients provided with 20 billion organisms from a probiotic drink [18]. In a German study conducted on 107 hospitalized patients, the incidence of AAD was significantly reduced in the intervention group (6.5% vs. 28.4%) and the duration of AAD was significantly shorter in the probiotic group (1.7 days vs. 3.1 days) [19]. This trial utilized the same commercially available probiotic drink providing 20 billion cfu/day of *Lactobacillus casei* DN 114001 as the Hickson trial.

In Oregon, the widespread usage of preventative probiotic beverages from yogurt or kefir has resulted in a 42–64% reduction in hospital acquired CDI when compared to historical CDI rates [20]. The incidence of CDI in Oregon hospitals that have implemented hospital-wide probiotic policies are considerably lower than national averages reported by the U.S. Centre for Disease Control [20].

Based on recent evidence, the Canadian Critical Care Guidelines upgraded their recommendation for probiotic usage in the ICU in 2013. The recommendation is that probiotics should be considered in critically ill patients for their modest treatment effect on reduction of infections [21]. Despite the heterogeneity of the systematic reviews and meta-analyses conducted over the past 10 years, they all conclude that probiotics are moderately effective in reduction of incidence of primary CDI [22]. The latest Cochrane systematic review has demonstrated that patients with the highest risk to develop diarrhea secondary to CDI have the greatest benefit from adjunctive probiotic therapy [14].

The abundance of research conducted to date has utilized pharmaceutical capsules, many of which are enteric-coated making administration by feeding tube difficult. Cost-effectiveness analysis of probiotics has been completed on medication based probiotics, however, to the authors’ knowledge, has never been completed on probiotic foods/beverages [23].

We conducted the trial to demonstrate that a probiotic beverage, readily available commercially in a Canadian setting, could be a viable option for probiotic delivery in our local ICU environment where all patients enrolled received nutrition by nasogastric or oral-gastric tube. Research has shown benefit of a similar probiotic treatment in a non-ICU setting in patients on oral diets but there is limited research to support the feasibility of a probiotic yogurt drink in the ICU [18,19]. There is medical consensus, as supported by numerous recent publications, to decrease antibiotic usage throughout hospitalization, making antimicrobial stewardship a top priority within acute and long term care facilities. Other risk factors for development of AAD and CDI include hospitalization, increasing age and multiple comorbidities [23].

The purpose of this study is to determine feasibility of delivering a probiotic yogurt as a prevention strategy of AAD in critically ill adults. Our primary goal is to determine safety and feasibility of administering a probiotic containing drink via gastric feeding tube or by addition to oral diet. The secondary outcome to be measured is the occurrence of AAD and CDI.

## 2. Methods

Population: The study population consisted of 32 ICU patients, matched with respect to age, BMI, and severity of illness. This pilot study aimed to recruit a convenience sample of ICU patients initiated on antibiotic therapy. Ethical approval to conduct the study was provided by the University of Alberta Health Research Ethics Board. The case-controlled trial attempted to follow the CONSORT guidelines and the STROBE statement for reporting parallel group randomized trials [24,25]. Patients were recruited from a single ICU in a 25-bed academic teaching hospital. The ICU research nurses identified patients that met study inclusion criteria, and offered participation to the study to immediate family members.

### 2.1. Inclusion Criteria for Patients to Be Offered Enrolment

- age > 18 years with immediate family able to provide written informed consent;
- prescribed one or more antibiotic in ICU;
- functional intact gastrointestinal tract; and
- anticipated ICU stay of >72 h after enrollment.

Exclusion criteria:

- received oral or IV antibiotics for >48 h in hospital;
- received a course of antibiotics in the past 30 days;
- history of CDI in previous 90 days;
- poor prognosis and not anticipated to survive the probiotic treatment period;
- one of the following medical diagnoses: immunosuppression, bowel resection, artificial heart valve, infective endocarditis, rheumatic heart disease, pancreatitis, or inflammatory bowel disease;
- permanent resident in long term care;
- known to regularly consume probiotics; and
- history of milk allergy or intolerance to dairy products.

During the study, the investigators adjudicated whether the diarrhea was secondary to antibiotics, promotility agents or disease-based. Each enrolled subject was matched with a contemporary control. The control patients were chronologically recruited at the same time as the probiotic patients were being enrolled. If they met all inclusion and exclusion criteria, they served as a study control. Reasons that patients were not included in the treatment group were that family was not available to provide consent or the time to potential consent was greater than 48 h. Interventions: Study patients received a probiotic yogurt drink, Danactive^®^ (Danone, Boucherville, QC, Canada) containing 10 billion cfu of *Lactobacillus casei* sp. Paracasei CNCM I-1518 (formally DN-114 001) [26]. This is the same product that demonstrated effectiveness in prevention of AAD in the Hickson trial [18]. The probiotic was stored as per industry recommendations. Once participants began antibiotic therapy, probiotic therapy was initiated within 48 h of receiving their first antibiotic. One container (93 mL) of the probiotic liquid was bolused twice daily when patients were being fed with a feeding tube. Administration times differed from antibiotic administration times by 2 h or greater when possible. The tube was flushed with 30 mL sterile water pre- and post-administration times, as per standard unit protocol for medications or supplements. All patients were fed continuously by enteral nutrition as per standard ICU protocol; nutritional assessment of energy and protein requirements, and enteral formula selection was determined by the ICU dietitian in conjunction with the medical staff. The ICU dietitian monitored the patients for nutritional adequacy and complications as part of routine care. 

The probiotic drink was provided twice daily to patients consuming an oral diet. Researchers, together with nursing staff, verified participant’s consumption and recorded missed or refused drinks to assess compliance. Once antibiotic therapy was completed, probiotic therapy continued for seven days while the patient remained in the ICU. Probiotic treatment was discontinued at the time of ICU discharge. Nutritional status and feeding tolerance was monitored by ward dietitians, as per usual nutrition care post ICU discharge. Demographic markers recorded included age, gender, admitting diagnosis, co-morbidities and severity of illness (APACHE II). All study data collected is listed in Table A1.

### 2.2. Outcome Markers that Were Measured Include

Incidence of diarrhea related to antibiotic administration while in the ICU—defined as three or more bowel movements per day or greater than 750 mL liquid stool—was determined retrospectively using the electronic medical record. When diarrhea continued for greater than three consecutive days, in conjunction with antibiotic administration, the diarrhea was defined as AAD.

Incidence of CDI was per laboratory confirmation by stool enzyme immunoassay for enterotoxin A or cytotoxin B produced by *C. difficile* bacteria.

Hospital acquired CDI was defined as presence of *C. difficile* toxins after 72 h of hospitalization. A positive CDI result that occurs within 72 h of hospitalization is defined as community-acquired CDI as defined by national policy [27]. The risk of antibiotics causing diarrhea was graded and included in the outcomes analysis. The percentage of assessed energy and protein was recorded daily, as enteral feed intake greater than 60% in combination with antibiotics in ICU has been previously cited as a risk factor for development of diarrhea [28].

Estimates of oral food consumed (0–50% OR 50–100%) was taken when patients were transitioned to an oral diet. The energy and protein from the probiotic drink was included in the calculation of energy/protein in the probiotic group. Use of parenteral nutrition was recorded but not included in the calculation of energy and protein intake. Gastrointestinal tolerance was assessed daily inclusive of incidence of diarrhea, and nausea and vomiting. Antibiotic usage and route were recorded daily. Concomitant use of anti or pro-motility agents, bowel routine medications and opioid use was recorded. The probiotic drink was continued for the length of time the patient received antibiotics in ICU, and for seven days post discontinuation of antibiotics if patient remained in ICU. If antibiotics discontinued and restarted within 48 h we resumed study probiotic treatment. If antibiotics stopped for >48 h, and restarted, no further study probiotic treatment was given beyond the seven days from the initial discontinuation of antibiotics or at ICU discharge (whichever was earlier). WBC, albumin and C-reactive protein were recorded if done as standard of care while on study treatment. All stool cultures done were monitored for a positive or negative finding of *Clostridium difficile* toxin A or B. Probiotic treatment was discontinued upon discharge from the ICU. Length of ICU and hospital stay was recorded. After the treatment period, subjects entered a follow-up period. During the follow up period, all subjects were evaluated for outcome markers at seven days from last dose of the probiotic drink.

Thirty days after final antibiotic dose, we reviewed subject’s paper and/or electronic health care records. We specifically noted stool culture results, incidence of diarrhea or other significant adverse events and any further antibiotic use. Significant adverse events related to dysfunction of the gastrointestinal tract were monitored. These included: insertion of rectal tube for diarrheal control, emesis, requirement for parenteral nutrition, and need for surgical management of GI tract.

Statistical Analysis: Fisher’s exact test was used to compare rates of AAD and CDI. Results were compared to case controls not receiving probiotic treatment. The acceptability of the product was monitored through researcher’s record of consumption of twice daily probiotic. With a two-sided alpha of 0.05 and a power of 80% to detect an absolute difference between the patients with AAD in the placebo (previously assessed at 30%) and probiotics, we estimated we needed a sample size of 300. This study aimed to enroll 40 patients to determine efficacy and feasibility of the protocol.

## 3. Results

The study population consisted of 32 ICU patients. Mean age of the study patients was 59.9 and 57.5 years in the probiotic and control groups, respectively. Mean APACHE II score was 25.5 and 25.88 in the probiotic and control groups, respectively. The majority of patients in both groups were non-surgical patients (Table 1).

Compliance to probiotic intake and adverse events from probiotics were documented throughout the study. Compliance with the probiotic drink was noted to be 92%, with NPO status serving as the main reason for non-compliance. All antibiotics were graded for their risk of developing diarrhea (Table 2). During the study, two investigators adjudicated whether the diarrhea was secondary to antibiotics, promotility agents or disease-based causes. In the event of a discrepancy, a third investigator was asked to adjudicate the cause of diarrhea.

Incidence of AAD and CDI was determined. Two patients (12.5%) in the treatment group developed AAD compared to five patients (31.3%) in the control group. The incidence of AAD was similar to previously defined AAD rates from unpublished quality review statistics of our ICU. Since the definitions of AAD are diverse and the incidence of diarrhea in ICUs is high, we chose the more stringent definition for this trial (three or more bowel movements for three days or more) [18,19]. One patient in the probiotic group developed CDI at Day 5 (6%) compared to three patients in the control group (18.75%) (Table 3).

There were three serious adverse events in the control group: two patients required rectal tubes for management of intractable diarrhea, one of these patients required a hemicolectomy and parenteral nutrition support for 23 days (eight days of PN alone and 15 days of combined PN/EN). There were three incidents of emesis in the probiotic group, which was adjudicated as secondary to probiotic administration.

The nutritional requirements of both groups were similar; energy needs were met in 73.9% of probiotic group and 68.1% of control group; 74.1% of protein requirements were met in the probiotic group compared to 69.8% of protein needs met in control group (Table 4). All patients were fed initially by EN, and then transitioned to oral diet later in their ICU admission. Number of days on EN and enteral formula selection were similar for both groups (Table 4). No differences were shown with respect to ICU and hospital survival or length of stay.

## 4. Discussion

We have shown that a probiotic containing drink can be safely delivered via naso/oralgastric feeding tube in the ICU, and is acceptable for oral consumption once patients transition to an oral diet. Three episodes of emesis that could be attributed to the administration of the probiotic drink were noted. This may be attributed to the high osmolality of a flavored commercial product of probiotic drink. The osmolality of fruit flavored yogurts has been estimated at 871 mOsm/kg compared to the osmolality of commonly used EN (300–650 mOsm/kg) [6,29]. A hospital based protocol implemented in Oregon uses an unflavored kefir product that is diluted 50:50 with water, and flushed with 30 mL water pre- and post-delivery [20]. An unflavored version of the commercial product used in our study is currently not available, but results may demonstrate the need for a diluted product, slower infusion time with the 93 mL product, and ideally an unflavored product.

Although no statistical significance was shown, the incidence of AAD in combination with high risk antibiotic administration is noteworthy. There was no difference in the incidence of CDI between the probiotic and control groups; one patient in the probiotic group developed CDI on Day 5 and did not experience any serious adverse effects; in contrast, three patients in the control group developed CDI and went on to develop severe adverse events: rectal tubes ×2 for intractable diarrhea, 23 days PN course for one patient and a hemicolectomy for the same patient. Two of these patients developed CDI in ICU while receiving antibiotics while the third patient developed CDI on Day 30 after transfer to the ward while continuing antibiotic treatment. There were no major adverse events in the probiotic group.

The trial was successful in recruiting 32 patients despite screening 850 patients over 25 months. Of all patients screened, 307 (36%) were ineligible due to intake of antibiotics over the previous 30 days. Although antibiotic stewardship is advocated, ICU patients are often medically treated for a variety of comorbidities prior to ICU admission, often involving initiation of antibiotics. We demonstrated safety and feasibility of a probiotic drink in enterally fed critically ill patients. The low recruitment rate demonstrates the need to begin probiotics earlier on surgical and medical wards, or in the community, when antibiotics are ordered. Due to the high prevalence of established antibiotic regimes in ICU patients, research into probiotics as secondary prevention post-antibiotics initiation needs to be explored. Although some adjustments may be required for enterally fed patients in dilution and rapidity of administration, probiotic beverages can be safely administered in appropriate patients and is an acceptable product for patient’s oral consumption. Patients consumed 100% of the oral probiotic offered independent of therapeutic or consistency diet modifications.

The strength of our study was the successful timing of the probiotic delivery to be within 48 h of antibiotic treatment commencing, and the exclusion of patients that had been on antibiotics in the month prior to probiotic initiation. Patients consistently received 2 doses/day of probiotic drink providing 20 billion *Lactobacillus casei* organisms/day with 92% compliance independent of whether receiving nutrition by tube or by mouth.

One of the limitations of the study is that the majority of ICU patients are exposed to antibiotics prior to ICU admission, making primary prevention of AAD and CDI more difficult to assess. As a pilot, the study was not powered to demonstrate the efficacy of a probiotic drink in preventing AAD or CDI. The study was case-controlled and was not blinded, and the decision to send stool for *C. difficile* analysis was practitioner dependent. An unflavored version of the study product is not available, and osmolality of the probiotic drink is not known and may have been a factor in the three incidents of emesis in the probiotic cohort. For logistic reasons, probiotic therapy was not continued post ICU discharge, and patients were not followed for incidence of diarrhea following ICU discharge. We did follow patients throughout their hospitalization for positive CDI results. Although our sample size was small, the incidence of AAD and CDI in our study is consistent with AAD and CDI rates in larger trials where statistical significance was achieved using this probiotic drink.

## 5. Conclusions

Previous Cochrane reviews and meta-analyses have concluded there is moderate quality evidence to suggest that probiotic prophylaxis results in a large reduction in diarrhea associated with CDI, without an increase in clinically important adverse events. This conclusion was drawn despite heterogeneity of probiotics used. Recent meta-analyses of probiotic effectiveness have demonstrated that probiotics are significantly more effective if given closer to the time of the first antibiotic dose.

The results of this trial have demonstrated that a *Lactobacillus casei* beverage can safely be added to EN of ICU patients. ICU patients have historically been excluded from probiotic trials in view of safety concerns, however, due to the large number of high risk antibiotics prescribed, along with the high incidence of AAD, these patients may have the most to gain. Although positive benefits of probiotics in ICU have been realized with a decrease in infectious episodes, including CDI, immunocompromised patients should continue to be excluded due to safety concerns [30].

Patients find the commercially available probiotic beverages acceptable and have demonstrated a willingness to consume while taking an oral diet. This pilot points to the need to assess a probiotic drink as standard adjunctive preventative therapy for all ICU patients at risk for AAD and CDI. Due to the routine exposure to antibiotics in ICU, the evaluation of probiotics as secondary prevention to patients already established on antibiotics needs further exploration. It appears that a *Lactobacillus* probiotic drink is feasible and safe for ICU patients, however, optimal dose and strain remains elusive. A cost-effectiveness analysis is required to determine if a commercially available probiotic drink added to patients’ oral diets or EN is efficacious and provides a viable alternative to probiotic supplements for the prevention of AAD and CDI in ICU and hospitalized patients.

## Figures and Tables

**Table 1:** Baseline Characteristics of critically ill patients.

Characteristics	Probiotic Intervention (*n* = 16)	Control Group (*n* = 16)
Age, year (mean ± SD)	59.9 ± 15.6	57.5 ± 15.0
Male sex, *n* (%)	12 (75)	10 (62.5)
BMI *, kg/m^2^ (mean ± SD)	25.0 ± 10.2	25.2 ± 6.0
Height, cm (mean ± SD)	172.6 ± 17.6	173.7 ± 9.9
Weight, kg (mean ± SD)	74.7 ± 22.9	75.5 ± 15.7
APACHE II score ** (mean ± SD)	25.5 ± 5.39	25.9 ± 9.7
Race, *n* (%)		
Caucasian/White	14 (87.5)	14 (87.5)
Black/African/African-American	1 (6.3)	0 (0.0)
Native	0 (0.0)	2 (12.5)
Hispanic	1 (6.3)	0 (0.0)
Admission category, *n* (%)		
Operative	3 (18.8)	1 (6.3)
Non Operative	13 (81.3)	15 (93.8)
Admission Diagnosis, *n* (%)		
Cardiac Arrest	1 (6.3)	0 (0.0)
Respiratory (Incl. pneumonia)	7 (44.0)	5 (31.2)
Sepsis	0 (0.0)	2 (12.5)
Pulmonary Edema	1 (6.3)	0 (0.0)
Trauma (Incl. Head)	4 (25.0)	4 (25.0)
Brain Injury (incl. Hemorrhage)	3 (6.3)	0 (0.0)
Other Medical Diseases (Hypernatremia, Smoke inhalation, Overdose, Diabetic Ketoacidosis, Meningioma)	0 (0.0)	5 (19.0)
Charlson comorbid condition, *n* (%)		
None	12 (75.0)	12 (75.0)
Angina	1 (6.3)	1 (6.3)
Valvular Disease	2 (12.5)	1 (6.3)
Myocardial Infarction	1 (6.3)	0 (0.0)
Chronic Heart Failure	0 (0.0)	1 (6.3)
Hypertension	0 (0.0)	1 (6.3)
Peripheral Vascular Disease	2 (12.5)	6 (37.5)
Asthma	7 (43.8)	3 (18.8)
Chronic Obstructive Pulmonary Disease	0 (0.0)	1 (6.3)
Dementia	1 (6.3)	0 (0.0)
Diabetes (I or II)	1 (6.3)	2 (12.5)
Diabetes (end organ)	2 12.5)	3 (18.8)
Renal Disease	0 (0.0)	1(6.3)
Gastrointestinal Reflux	4 (25)	3 (18.8)
Any Tumour	1 (6.3)	2 (12.5)
Arthritis	2 (12.5)	3 (18.8)
Visual Impairment	1 (6.3)	1 (6.3)

* Body Mass Index ** Acute Physiology and Chronic Health Evaluation score.

**Table 2:** Medication Use during ICU Admission.

	Probiotic Intervention (*n* = 16)	Control Group (*n* = 16)	*p* Value
Laxative Use, *n* (%)	7 (43.8)	13 (81.3)	0.066
Senna	5 (31.3)	6 (37.5)	1.00
Colace	5 (31.3)	4 (25.0)	1.00
Dulcolax	10 (62.5)	6 (37.5)	0.289
Fleet	6 (37.5)	5 (31.3)	1.00
Peg3350	9 (56.3)	11 (68.7)	0.716
Antibiotic Type, *n* (%)			
Cephalosporins (Frequently Associated with AAD)			
Cefazolin	7 (43.7)	7 (43.7)	1.000
Cefuroxime	2 (12.5)	2 12.5)	1.000
Ceftriaxone	14 (87.5)	11(68.7)	0.394
Cefixime	2 (12.5)	0 (0.0)	0.484
Cephalexin	1 (6.2)	0 (0.0)	1.000
Penicillins (Frequently Associated with AAD)			
Penicillin G	0 (0.0)	1 (6.2)	1.000
Amoxicillin	4 (25.0)	0 (0.0)	0.101
Piperacillin/Tazobactam	7 (43.7)	7 (43.7)	0.394
Fluoroquinolones (Frequently Associated with AAD)			
Ciprofloxacin	0 (0.0)	2 (12.5)	0.484
Levofloxacin	1 (6.2)	0 (0.0)	1.000
Macrolides (Occasionally Associated)			
Azithromycin	6 (37.5)	7 (43.7)	1.000
Carbapenem (Occasionally Associated with AAD)			
Meropenem	0 (0.0)	2 (12.5)	0.484
Imipenim/cilastatin	0 (0.0)	1 (6.2)	1.000
Other (Rarely Associated with AAD)			
Metronidiazole	8 (50.0)	5 (31.2)	0.473
Voriconazole	1 (6.2)	0 (0.0)	1.000
Vancomycin	0 (0.0)	3 (18.7)	0.226
Number of Antibiotics received during study period, *n* (%)			
1	0 (0.0)	0 (0.0)	
2	2 (12.5)	3 (18.1)	
3	4 (25.0)	5 (31.3)	
4	6 (37.5)	1 (6.2)	
5	1 (6.2)	5 (31.3)	
6	2 (12.5)	1 (6.2)	
7	1 (6.2)	1 (6.2)	
Number of Antibiotics received that are frequently associated with diarrhea (High Risk), *n* (%)			
0	0 (0.0)	1 (6.2)	
1	5 (31.3)	5 (31.3)	
2	4 (25.0)	5 (31.3)	
3	5 (31.3)	5 (31.3)	
4	1 (6.2)	0 (0.0)	
5	1 (6.2)	0 (0.0)	
≥1 High Risk Antibiotic, *n* (%)	16 (100.0)	15 (93.8)	
High Risk antibiotic and AAD	2 (37.5)	3 (18.1)	
Independent Sample T-Test			
Number of Antibiotics received, mean ± SD	4.0 ± 1.41	3.94 ± 1.53	0.905
Number of High risk Antibiotics received, mean ± SD	2.31 ± 1.20	1.88 ± 0.96	0.350
AAD + high risk antibiotic received, mean ± SD	0.13 ± 0.34	0.25 ± 0.45	0.076

**Table 3:** Outcome Data.

	Probiotic Intervention (*n* = 16)	Control Group (*n* = 16)	*p* Value
Diarrhea			
Diarrhea *n* (%)	11 (68.8)	10 (62.5)	1.000
No Definite Cause	2 (12.5)	0 (0.0)	
Antibiotic Associated Diarrhea, *n* (%)	2 (12.5)	5 (31.3)	0.394
Laxatives Cause, *n* (%)	5 (31.3)	3 (18.7)	0.685
Outcome Diarrhea (Caused by AAD * or CDI *)	3 (18.7)	7 (44.0)	0.252
Proportion of Consecutive Diarrhea Days ≥2, *n* (%)	5 (31.3)	8 (50.0)	0.473
Diarrhea (rate over 100 patient days)	19/100	24/100	
Diarrhea Duration post AAD * diagnosis, days mean ± SD	1.0 ± 2.73	1.44 ± 2.99	0.574
Total Diarrhea Days, mean ± SD	2.13 ± 2.8	3.69 ± 4.44	0.241
CDI			
ICU Acquired *Clostridium difficile*, *n* (%)	1 (6.0)	2 (12.5)	0.600
Community Acquired *Clostridium difficile*, *n* (%) **	1 (6.0)	0 (0.0)	1.000
30 Day Outcomes			
30 Day Survival, *n* (%)	13 (81.3)	12 (75.0)	1.000
30 days outcomes CDI ***	1 (6.3)	3 (18.8)	0.600
30 days outcomes AAD	2 (12.5)	5 (31.3)	0.394
30 days outcome CDI or AAD	3 (18.8)	6 (37.5)	0.433
Survival and Length of Stay			
ICU Survival, *n* (%)	15 (93.7)	14 (87.5)	1.000
Hospital Survival, *n* (%)	14 (87.5)	14 (87.5)	1.000
Wilcoxon Signed Ranks			
ICU Length of Stay, Days mean ± SD	11.38 ± 7.4	15.31 ± 12.96	0.300
Hospital Length of Stay, Days mean ± SD,	79.56 ± 116.8	39.38 ± 54.74	

* AAD (Antibiotic Associated Diarrhea); CDI (*Clostridium difficile* Infection); ** Community acquired CDI defined as positive CDI within 72 h of hospital admission; *** 30 days outcome CDI includes patients who developed CDI post-ICU discharge until Day 30 of hospitalization.

**Table 4:** Nutrition Data during ICU Admission.

	Probiotic Intervention (*n* = 16)	Control Group (*n* = 16)	*p* Value
Average Energy Prescribed (kcal) mean ± SD	1944.5 ± 354.4	2068.8 ± 973.6	0.636
Average Energy Intake, kcal mean ± SD	1436.9 ± 414.2	1408.8 ± 352.4	0.837
Average Protein Prescribed (g) mean ± SD	117.5 ± 20.6	109.6 ± 27.9	0.371
Average Protein Intake, g mean ± SD	90.66 ± 34.1	79.1± 27.4	0.298
Average Fiber Intake, g mean ± SD	10.9 ± 5.2	10.6 ± 5.5	0.884
Enteral Nutrition, days mean ± SD	10.4 ± 7.5	11.0 ± 6.64	0.824
Oral Nutrition, days mean ± SD	3.5 ± 4.3	3.2 ± 4.4	0.840
Oral Nutrition >50% mean ± SD	1.38 ± 0.7	2.3 ± 3.2	0.299
Total Parenteral nutrition, days mean ± SD	0.0	1.44 ± 5.75	0.325
Total Parenteral nutrition/NPO *, days mean ± SD	0.0	0.50 ± 2.00	0.325
Days of Probiotic, mean ± SD	10.31 ± 4.2	----	
Enteral Nutrition Products	*n* = 16	*n* = 16	
Isosource Fiber, *n* (%)	2 (12.5)	0 (0.0)	
Isosource VHN ** *n* (%)	10 (62.5)	10 (62.5)	
Isosource 1.5, *n* (%)	2 (12.5)	3 (18.0)	
Resource Diabetic, *n* (%)	1 (6.3)	3 (18.0)	
Novosource Renal, *n* (%)	1 (6.3)	2 (12.5)	
Peptamen 1.0, *n* (%)	1 (6.3)	1 (6.3)	
Peptamen AF 1.2, *n* (%)	4 (25.0)	3 (18.0)	
Peptamen 1.5, *n* (%)	2 (12.5)	2 (12.5)	
Peptamen Intense, *n* (%)	2 (12.5)	3 (18.0)	

* NPO (Nil per os); ** VHN (Very High Nitrogen).

## Appendix A

**Table A1:** Tool used for Data Collection.

	Baseline	Day 1 to End of Probiotic Treatment	7 Days Post End of Probiotic Treatment	Hospital Discharge	30 Days Post Antibiotic Stop Follow up Visit
Informed Consent	x				
Gender/Race	x				
Age (years)	x				
Height/Weight/BMI ^1^	x				
Co-morbidities	x				
Albumin	x	x			
C-Reactive protein	x	x			
White Blood Cells	x	x			
Disease Severity Score (APACHE II ^2^)	x				
Length of Stay	x			x	
Gastro-intestinal adverse events		x			
Number and Route of antibiotics		x	x	x	x
Indication for antibiotics		x	x	x	x
Risk of diarrhea	x	x			
Stool cultures	x	x	x	x	x
EN/po ^3^ energy prescription (kcals/day)		x			
EN/po protein prescription (g/day)		x			
EN/po energy intake (kcals/day) ^4^		x			
EN/po protein intake (g/day) ^4^		x			
Fiber intake (g/day)		x			
Supplemental Parenteral Nutrition required (Y or N); Reason		x	x		
Weight weekly	x				
Additional probiotic use			x	x	
Volume of Danactive taken (mL/24 h)		x			
Bowel Movements per 24 h/CDI (Y or N)		x	x	x	x
Concomitant medications ^5^	x	x			

^1^ BMI (Body Mass Index); ^2^ APACHE II (Acute Physiology and Chronic Health Evaluation score); ^3^ EN (Enteral Nutrition); po (oral intake); ^4^ Energy/protein intake will be an estimate only when on oral diet. ^5^ Use of anti- or pro-motility agents, bowel routine medications and opioid use.
